# Strongyloidiasis Current Status with Emphasis in Diagnosis and Drug Research

**DOI:** 10.1155/2017/5056314

**Published:** 2017-01-22

**Authors:** Tiago Mendes, Karen Minori, Marlene Ueta, Danilo Ciccone Miguel, Silmara Marques Allegretti

**Affiliations:** Department of Animal Biology, Post-Graduation Program in Animal Biology, Institute of Biology, University of Campinas (UNICAMP), Campinas, SP, Brazil

## Abstract

Strongyloidiasis is a parasitic neglected disease caused by the nematode* Strongyloides stercoralis* affecting 30 to 100 million people worldwide. Complications, strongly associated with alcoholism, organ transplants, and HTLV-1 virus, often arise due to late diagnosis, frequently leading to patient death. Lack of preemptive diagnosis is not the only difficulty when dealing with this parasite, since there are no gold standard diagnostic techniques, and the ones used have problems associated with sensitivity, resulting in false negatives. Treatment is also an issue as ivermectin and benzimidazoles administration leads to inconsistent cure rates and several side effects. Researching new anti-*Strongyloides* drugs is a difficult task since* S. stercoralis* does not develop until the adult stages in* Mus musculus *(with the exception of SCID mice), the main experimental host model. Fortunately, alternative parasite models can be used, namely,* Strongyloides ratti* and* S. venezuelensis.* However, even with these models, there are other complications in finding new drugs, which are associated with specific in vitro assay protocol steps, such as larvae decontamination. In this review, we highlight the challenges associated with new drug search, the compounds tested, and a list of published in vitro assay methodologies. We also point out advances being made in strongyloidiasis diagnosis so far.

## 1. Introduction

Strongyloidiasis, caused by nematode parasites of the genus* Strongyloides*, is a cosmopolitan neglected disease with high prevalence in Caribe, Latin America, Europe, Asia, and sub-Saharan Africa [1]. In Humans,* Strongyloides stercoralis*, which can also parasite other mammals such as dogs or cats, is the predominant species [2, 3]. An estimated 30 to 100 million people are infected worldwide, though it is believed that this number is underestimated due to inappropriate or low sensitivity diagnostic techniques [3].

Several studies have been published using murine models focusing in immunodiagnostic or in the host immune response, in an attempt to understand immunological aspects that are still unknown [4–10]. Immunosuppression has been correlated with the infection aggravation, mainly in cases of HTLV-1 virus coinfection, alcoholism, and corticoids use [11–14]. Understanding the parasite behavior in this particular context will allow not only the research and development of better therapeutic options but also better diagnostic methodologies that could lead to detection of lower parasite burden infections, allowing patients to be treated before their health deteriorates.

Ivermectin is the drug of choice against* S. stercoralis*; however, its use is restricted in several countries, and a consensus, regarding how many dosages should be given or the time between each dosage, has not been reached. Ivermectin also shows irregular cure rates, varying between 55 and 100% in immunocompetent patients [29–31]. Other drugs, such as albendazole (cure rates between 38 and 87%) and its analogues have also been used, with thiabendazole being the most commonly used, with cure rates between 52 and 100%, though its use may cause side effects such as nausea, vomit, anorexia, asthenia, and diarrhea along with neurologic effects such has dizziness, sleepiness, and disorientation, making its use discouraged by health professionals [29, 31].

In this review, we highlight the need to develop new diagnostic techniques with higher sensitivity and the importance of mandatory* S. stercoralis* screening not only for organ donors and receivers but also for immunosuppressed patients in general. Besides, more efficient treatment strategies are lacking as options for the management of the strongyloidiasis patient, requiring the establishment of more adequate dosages and the research of new drugs/drug formulations. Then, a list of the drugs being searched and the methodologies employed are summarized in the present review.

## 2. *S. stercoralis* Immunosuppression and Diagnosis

HTLV-1 is a RNA retrovirus belonging to the Retroviridae family prevailing in Japan, sub-Saharan Africa, Melanesia, Middle East, and South America. Associated with T-cell lymphoma, HTLV-1 has been correlated with* S. stercoralis* infection since it depresses the host's immune system allowing the parasite to establish the infection [32–37].

Upon entering T-cells, the lymphotropic virus intensifies cell production, generating an exacerbated Th1 response, with high levels of INF-*γ*, TNF-*β*, and IL-2, along with a decline in IL-4, IL-5, and IL-10 production [38, 39]. Viral-caused immunosuppression promotes massive* S. stercoralis* infections, making treatment difficult due to parasite dissemination. Among reported cases, the most common symptomatology associated with this coinfection consists in vomits, diarrhea, weight loss, cough, and, in some cases, larvae and/or adult females can be found in sputum [14, 32–36, 39, 40].

Alcoholism is also associated with* S. stercoralis* infections [13, 41]. Ethanol depresses the immune system and, when excessively consumed, causes behavioral changes, which can further expose an already impaired host to infections [41]. In a paper published by Silva et al. [13],* S. stercoralis* infection prevalence was higher in alcoholic patients (23.5%) than in nonalcoholic ones (5.4%). Among the alcoholic patients, 81.3% presented high parasite loads, suggesting that high cortisol levels, commonly associated with alcoholism, may lead to massive infections. Increase in ethanol levels leads to an increase in corticosteroids, reducing T-cells function and increasing* S. stercoralis* females fertility [13], alerting that not only alcoholic patients but also patients requiring corticosteroids use are at risk.

There are several reported cases of organ-receiving patients, with daily corticosteroids use, suffering complications due to* S. stercoralis* infection, often leading to their death [42–56]. Usually, in transplanted patients, strongyloidiasis diagnosis is only revealed after complications appear, pointing to a need of preemptive diagnosis before the transplant, not only of the recipient, but also of the donor, since cases of passage through infected organs have been described [57]. In a paper publish by Luvira et al. [58] with 135 immunocompromised patients, using 3 diagnostic techniques, 8 were infected with* S. stercoralis, *where* Strongyloides* IgG detection through indirect enzyme-linked immunosorbent assay (ELISA) only showed a 42,9% sensitivity. Steinmann et al. [59] also observed that, in cases where treatment failed but parasite load reduced, the diagnostic tests often failed to detect remaining parasites, showing a requirement for more sensitive tests.

Due to* S. stercoralis* ability to autoinfect the host, only complete cure eliminates the risk of future complications; therefore, it is mandatory to employ accurate diagnostic methodologies capable of detecting light and mild infections [60]. Currently, there is no gold standard diagnostic technique for* S. stercoralis *[61, 62]. Routine diagnosis usually consists of parasitological and/or serological methods, though their performance is far from great [60]. Several parasitological tests have been used to detect larvae in stool samples, including Baermann method, formalin-ethyl acetate, Harada-Mori culture, and Agar Plate Culture (ACP); however, these tests have low sensitivity (21 to 89%) due to low parasite load and irregular larvae elimination, making it necessary to collect several stool samples throughout several days [7, 60, 63]. Among these methods, ACP is considered the most efficient but also the most laborious one, taking 2 to 3 days, requiring fresh stool samples and an experienced technician [7, 63–65].

Several immunologic techniques have been studied, including enzyme-linked immunosorbent assay (ELISA), indirect agglutination, indirect immunofluorescence, and western blot, all with different sensitivities and specificities, depending on the antigen and isotopic antiglobulin preparation [6, 9, 66–78]. Immunologic methods show a higher sensitivity than the conventional parasitological ones; nevertheless, there are concerns with its specificity due to cross-reactions with other [6, 67, 68] helminthes and with false positives due to antibody persistence. Although there are concerns regarding immunologic tests specificity, some works have shown promising results in the use of in-house indirect immunofluorescence antibody test (IFAT) (sensitivity 97%; specificity ~100%) or ELISA (sensitivity >97%; specificity ~100%) [60, 72, 79]. Even so, further studies are needed to optimize their use. It should also be noticed that, in immunosuppressed patients, immunodiagnosis can be tricky, due to reduced sensitivity of serum antibody detection, where the more immunocompromised the patient, the lower the sensitivity [60, 80]. Serological and epidemiological studies based on the diagnosis of human strongyloidiasis usually use both serology and stool culture, while some researchers have started to use PCR (conventional or not), though not so frequently [80].

Among diagnostic techniques, the utilization of polymerase chain reaction (PCR) (conventional PCR (cPCR), nested-PCR, or real-time-PCR (qPCR)) [7, 8, 62, 79–91] has been considered as a valuable method. PCR methodologies show superior sensitivity when compared with parasitological or immunological methods even studies indicating that sensitivity can vary, possibly as a result of the extraction method [7, 62, 79, 80, 82, 86, 88, 89]. Thus, optimizing and standardizing DNA extraction methodology are extremely important to increase molecular diagnostic sensitivity.

QPCR-based diagnosis has been more often applied, showing high sensitivity and specificity. Its use may represent a decrease in the amount of time needed for a diagnosis and also increase the parasite detection success, being used not only for fecal samples but also for cerebrospinal fluid samples from patients with* Strongyloides* associated meningitis [8, 62, 75, 79, 83, 84, 87, 88, 91]. Currently, qPCR is the most exploited molecular method due to its higher sensitivity comparing to cPCR (sensitivity 76,7%; specificity 84,3%) [84], but it also presents higher costs than the ones associated with cPCR. Sharifdini et al. [62] found a higher sensitivity in nested-PCR (sensitivity 100%, specificity 91.6%) compared to qPCR (sensitivity 84.7%, specificity 95.8%), representing a possible alternative with lower costs.

Better diagnostic tools for* S. stercoralis* infections are needed. Parasitological methods show an unsatisfactory sensitivity, even when APC is performed and although serological diagnosis shows a good sensitivity, there are several concerns regarding cross-reactions [6, 7, 60, 67–73, 75, 76]. Molecular diagnosis can be considered a promising tool that has been employed in some research and clinical laboratories, but with no standardized methodology so far [12, 40, 92–95]. Optimization of* S. stercoralis* diagnostic is certainly a crucial step in the fight against strongyloidiasis, so that future complications and/or fatalities caused by this parasite may be avoided.

## 3. Models for* Strongyloides *Infection in Preclinical Investigation and Drug Research

Treating* Strongyloides* infection represents a major challenge. Ivermectin is the most effective drug against* S. stercoralis*; however, it is not licensed in several countries and the treatment regimen still needs to be better assessed [29, 30]. Although albendazole and other benzimidazoles can be used as alternatives, their efficiency is inferior to ivermectin [30], showing an imperative need in investing and finding alternative anti-*Strongyloides* drugs.

Historically,* Mus musculus *mouse has been the most commonly used model to understand basic biology and diseases in Biomedical Research. Nevertheless, mice and rats are not susceptible to* S. stercoralis* infection [2, 96–98] since larvae penetrate the skin and migrate to the lungs, moving towards skeletal muscle, not being able to promote a patent infection or not even reaching the intestines, therefore making it impossible to maintain the parasite and quite challenging to be used in the laboratory routine [2, 97, 99]. It is interesting to note that, just like in immunocompetent mice, in nude mice (T-cell deficient),* S. stercoralis* does not reach maturity in the small intestine. However, in SCID mice (T- and B-cell deficient), the entire* S. stercoralis* cycle can be reproduced, though their high cost and special needs due to a compromised immune system limit its use [96].

*Strongyloides ratti*, a rat parasite, was the first alternative model to be used as a research tool in strongyloidiasis and, years later,* Strongyloides venezuelensis* was also acknowledged as suitable model for this disease. Although there are some limitations in utilizing infections by these species, mainly due to the absence of autoinfection (present in* S. stercoralis*),* S. ratti* and* S. venezuelensis* naturally infect rodents and also possess an endogenous and exogenous cycle, being important models to understand strongyloidiasis biology and immunology and in searching for new drug alternatives against* Strongyloides* [100–103].

Dogs and primates are naturally infected with* S. stercoralis *[104]. Up until the 90s, due to the lack of rodent models, most laboratories used them as experimental* S. stercoralis* hosts, studying the parasite biology and immunology [19]. Even though nowadays some laboratories still use them whether for parasite maintenance or laboratory experiments [18, 104], their use has decreased when Nolan et al. [23] published a work, proving that gerbils (*Meriones unguiculatus*) were able to develop a chronic infection similar to that observed in humans. Gerbils develop long course infections (~130 days) and autoinfection can be induced with steroid treatment, leading to massive hyperinfection, usually associated with human immunosuppression, making the gerbil an important model to study many biological and immunological aspects of the host-parasite interaction [18, 19, 23].

When researching new anti-*Strongyloides *candidates, some requirements need to be considered in order to ensure the quality of the infection, as the use of decontaminated larvae, which may be a challenge since they are obtained from feces [15]. Given the importance of larvae decontamination for the infection procedure, protocols regarding this step are summarized in Table 1.

The same can be mentioned for in vitro compound and drug testing assays. Currently, few anti-*Strongyloides* new drug research papers have been published; however, considering the small number, there is a great diversity of protocols used. Parameters evaluated in these papers include larvae survival, determined by parasite movement, and egg hatching (Table 2) [16, 17, 22, 24, 25]. A few research groups showed promising in vitro results, as the activity of 2-(butylamino)hexadecan-1-ol,* Newbouldia laevis *essential oil, and* Zanthoxylum zanthoxyloides *essential oil against* S. ratti *and* S. venezuelensis *or* Eryngium foetidum *crude extract and Eryngial (trans-2-dodecenal) against* S. stercoralis *[16, 21, 22] (Table 3). On the other hand, in vivo published data (summarized in Table 4) do not necessarily support results obtained in vitro. For example, in a study conducted by Legarda-Ceballos et al. [16], out of four compounds with promising in vitro results, only one was capable of reducing mice parasite load in ~60%. Compounds derived from* Piper tuberculatum*, with known insecticide, trypanocidal, and fungicidal action, and compounds derived from* Lippia sidoides*, effective against some bacteria and* Aedes aegypti *larvae, were ineffective against* S. venezuelensis *in vivo. Monepantel (AAD 1566), which has been used as an anthelminthic in veterinary medicine, was inefficient against* S. ratti *in vivo [27, 28]. Tribendimidine showed efficiency against* S. ratti *in vivo when applied in a single dosage of 50 mg/kg 72 h after infection, reducing the infection in 98.9% [17]; however, when used in a clinical trial, in a population infected with* S. stercoralis, *cure rates dropped by 54,5% [105]. Taking the data summarized into consideration, it is evident that laboratorial parasite models are extremely important although some limitations may arise when crossing over to* S. stercoralis. *An alternative to reduce these obstacles would be the use of gerbil* (Meriones unguiculatus), *a rodent model susceptible to* S. stercoralis *infection that shows autoinfection when experimentally immunosuppressed [23]. Unfortunately, using gerbils as in vivo model of infection for drug assays is still very limited; until recently, only one study has demonstrated the efficacy of ivermectin in these* S. stercoralis*-infected animals [19].

## 4. Concluding Remarks

Strongyloidiasis is a cosmopolitan neglected disease, whose complications are strongly associated with alcoholism, organ transplants, HTLV-1 virus, and immunosuppression in general [11–14]. It is important to highlight that, even with early diagnostic, problems associated with the tests sensitivity may lead to false negatives, raising a need for new diagnostic methodologies. Some diagnostic methods (mostly immunological and molecular) are under investigation and show great potential, but more studies are needed before they can be considered as alternatives to be available in the market [6, 63, 67, 79, 81]. Treatment is potentially difficult and unsuccessful due to ivermectin irregular cure rates and lack of consensus regarding the number of dosages and amount of time between each administration [29–31]. New drug research is a challenge since* S. stercoralis* does not infect the most commonly used laboratory models,* Mus musculus* and* Rattus norvegicus* [98]. Using* S. ratti* and* S. venezuelensis* has represented an important alternative in this area; however, self-healing and lack of standardized methods used in these assays seem to limit the number of alternative therapy studies over the years [10, 97, 98, 100].

In this review, we pointed out the great need of strongyloidiasis early diagnosis and some promising new diagnostic methods that are being under investigation. Besides, we summarized different protocols used in new drug research and which compounds have been tested both in vitro and in vivo, in an attempt to shed some light on preclinical research directed towards the discovery of novel anti-*Strongyloides* candidates.

## Figures and Tables

**Table 1:** Protocols used for *Strongyloides* spp. L3 larvae decontamination.

Species	Procedure	Confirmation of axenic state	Larvae use	Reference
*S. venezuelensis*	(i) L3 larvae were collected from Bearman culture (ii) Larvae were washed in 0.25% sodium hypochlorite solution for 10 min (iii) Larvae were exposed to benzylpenicillin (180 mg/L) and ceftazidime (1 mg/mL) (30 or 60 min)	Larvae were incubated at 25°C and 37°C for 7 days in tubes containing thioglycolate broth with brain and heart infusion	NIH germ-free mice infection	[15]

*S. venezuelensis*	(i) L3 larvae were collected from Bearman culture (ii) Larvae were washed 6 times for 20 minutes each in distilled water containing penicillin (100 IU/mL), streptomycin (0.1 mg/mL), and fluconazole (0.8 mg/mL)	Maintained in blood agar culture for 24 h at 28°C	96-well plate assay containing water	[16]

*S. ratti*	(i) L3 larvae were washed 3 times in PBS buffer	—	6-well plate assay containing 4 mL PBS buffer (pH 7.3)	[17]

*S. stercoralis*	(i) A concentrated suspension of L3 larvae was placed in a water bath at 37°C. (ii) 1 volume of low melting point agarose (3% low melting point agarose in BU saline; 50 mM Na_2_HPO_4_; 22 mM KH_2_PO_4_; 70 mM NaCl) at 37°C was mixed with 2 volumes of concentrated parasite suspension (this operation was performed with tubes in the water bath at 37°C to avoid solidification). (iii) A 100 mm Petri dish(s) was placed on ice, and 5 mL of parasite suspension in 1% agarose pipetted into the center of the plate avoiding contact with the sides of the plate (iv) After solidification, 10–20 mL of BU saline at 37°C was added to the plate and incubated at 37°C for 30 min Viable worms migrate from the agar matrix into the surrounding medium during this period (v) The liquid medium was then collected and transferred to a 15 mL conical centrifuge tube Worms can sediment by either gravity for 15 min or by centrifugation at 500 rpm for 5 min	—	In vitro assays	[18]

*S. stercoralis*	(i) L3 larvae were collected from agar culture (ii) Larvae were washed several times in saline solution (PBS) with penicillin (100 U/mL) and streptomycin (0.1 mg/mL)	—	Gerbil (*Merionesunguiculatus*) infection	[19]

*S. venezuelensis *and* S. ratti*	(i) L3 larvae were incubated at room temperature in water with Amphotericin B (0.25 *μ*g/mL) and ceftriaxone (20 *μ*g/mL)	—	96-well plate assay with melted agar at 1%	[20]

*S. stercoralis*	(i) L3 larvae were washed two times in M9 buffer (ii) Supernatant was removed and worm pellet was resuspended in the residual volume (iii) The worms were added to a conical centrifuge tube containing Percoll solution at 40% supplemented with 25 mM Heppes and 10,000 U penicillin/10 mg streptomycin (iv) The suspension was centrifuged at 1000 rpm for 2 min and washed twice with M9 buffer	—	In vitro assays	[18]

*S. stercoralis*	(i) L3 larvae were collected from charcoal cultures and washed by centrifugation and resuspension in sterile RPMI with 100 U/mL penicillin, 0.1 mg/mL streptomycin, and 0.1 mg/mL gentamicin (ii) Afterwards, they were placed for 20 min in a 2% solution of low-melt agarose, and PBS (supplemented with 100 U/mL penicillin, 0.1 mg/mL streptomycin, and 0.1 mg/mL gentamicin) was added after agarose solidification (iii) Larvae that migrated into the PBS solution were then harvested	—	L-3 antigen solubilization	[21]

“—”: not performed or not shown.

**Table 2:** Protocols used for in vitrodrug/compound tests.

Species	Purpose	Procedure	Reference
*S. venezuelensis*	L3 larvae motility and viability observation	(i) 100 L3 decontaminated larvae were added to water in 96-well plates. (ii) Compounds were added and plates were incubated at 28°C for 72 h. (iii) Larvae viability was quantified through the XTT colorimetric method. and by monitoring their movement (registered through exposure to light for 2 min using an inverted microscope and a video camera). (iv) Observations were made at 24, 48, and 72 hours after treatment. (v) Larvae were considered dead when no movement was detected after 2 min of observation.	[16]

*S. ratti*	Compounds with egg hatching effect	(i) Feces were homogenized in water and filtered through sieves with 250, 125 e 40 *μ*m. (ii) Eggs were collected and centrifuged (5 min, 3,500 ×g). (iii) Supernatant was removed and NaCl solution (350 g/L) was added. (iv) The mixture was homogenized and then centrifuged (5 min at 3,500 ×g).	[22]
		(v) The supernatant was filtered through a 40 *μ*m sieve and washed with water for egg extraction. (vi) Eggs were distributed in 96-well plates (100 eggs per well, 200 *μ*L final vol.). (vii) Plates were incubated at 23°C for 48 h. (viii) Incubation was stopped and formaldehyde solution (10% in PBS) was added. (ix) L1 larvae and eggs were counted from 200 *μ*L aliquots and hatched percentage was calculated.	

*S. ratti*	Compounds affecting L3 larvae migration	(i) L3 larvae were incubated for 3 h at 20°C in PBS solution mixed with the extracts. (ii) Larvae were washed 3 times in PBS solution and centrifuged. (iii) 800 *μ*L of larvae suspension at a concentration of 1000 L3/mL was placed over a 20 *μ*m filter. (iv) The filter was added to a conic tube in contact with the PBS solution surface. (v) After 3 h, larvae above the filter were discarded and the ones that actively penetrated through the mesh to the PBS were counted using optical microscope and the migration percentage was calculated.	[23]

*S. ratti*	Compounds with larvicidal activity in L3 larvae	(i) L3 larvae were washed 3 times in PBS and incubated in 6-well plates containing 4 mL PBS solution. (ii) Compounds were added and larvae were incubated for 96 h (25°C, 5% CO_2_). (iii) Observations were made at 1, 2, 24, 48, 72, and 96 h using optical microscope.	[17]

*S. stercoralis*	Drugs affecting L3 larvae motility	(i) ~1 g of feces was smeared at the center of a piece of filter paper and added in a glass tube. 5 mL of the tested drug was added to the bottom of the tube incubated at 25°C. (ii) The tubes were analyzed at the 3rd and 5th days to assess drug effect on worm viability through their motility.	[24]

*S. stercoralis*	Compounds affecting L3 larvae mortality	(i) Dog feces were added to agar plates and left at room temperature for 3 to 5 days. (ii) L3 larvae that migrated to the plate lid were collected, washed in saline solution (0,85%, pH 7.4), and centrifuged (2000 rpm, 5 min). (iii) Pellet was washed 3 times in PBS (0.1 M, pH 7.4) with penicillin G (1000 IU) and streptomycin (0,1 mg/mL). (iv) 1 mL of larvae suspension was added to 1 mL of the testing compound at a final concentration of 1,000 larvae/mL. (v) Plates were incubated at 37°C in a 5% CO_2 _ atmosphere. (vi) Mortality rate was assessed through larvae motility after observation under optical microscopy for seven days.	[25]

*S. stercoralis*	Compounds affecting L3 larvae mortality	(i) 50 L3 larvae in Locke's nematode saline (LNS) suspension containing plant extract (50 mg/mL) and eosin (0.1 mg/mL) were kept in a light-free environment between counts. (ii) The larvae were assessed as mobile, immobile (not stained), and dead (stained with eosin). (iii) Relative activity (RA) was calculated by dividing LT50 of the most effective extract (shortest time to kill 50%) by the LT50 of each extract.	[26]

**Table 3:** Drugs/compounds used in in vitro assays against *Strongyloides* spp.

Species	Compound	LD50/IC50/RA^*∗*^	Criteria	Reference
*S. venezuelensis*	2-(Butylamino)hexadecan-1-ol	LD50 = 52 *μ*M	L3 larvae death	[16]
	2-(Ethylamino)hexadecan-1-ol	LD50 = 52 *μ*M		
	*tert-*Butyl *N*-(1-aminododecan-2-yl) carbamate	LD50 = 39 *μ*M		
	*tert*-Butyl *N*-(1-aminohexadecan-2-yl) carbamate	LD50 = 39.1 *μ*M		

*S. ratti*	*Zanthoxylum zanthoxyloides *essential oil	IC50 = 19.5 *μ*g/mL	Egg hatching inhibition	[22]
		IC50 = 46 *μ*g/mL	L3 larvae migration inhibition	
	*Newbouldia laevis *essential oil	IC50 = 18.2 *μ*g/mL	Egg hatching inhibition	
		IC50 = 36 *μ*g/mL	L3 larvae migration inhibition	

*S. ratti*	Tribendimidine	100% L3 larvae death at 10 *µ*g/mL (24 h)	L3 larvae death	[17]

*S. stercoralis*	*Cardiospermum halicacabum *aqueous extract	LD50 = 2,000 *µ*g/mL (<24 h)	L3 larvae survival	[25]
	*Cardiospermum halicacabum *ethanolic extract	LD50 = 2,000 *µ*g/mL (>36 h)		

*S. stercoralis*	*Eryngium foetidum *crude extract	RA = 1.00	L3 larvae death	[26]
	*Portulaca oleracea *crude extract	RA = 0.606		
	*Cuscuta americana *crude extract	RA = 0.452		
	*Tamarindus indica *crude extract	RA = 0.437		
	*Bidens pilosa *crude extract	RA = 0.414		
	*Picrasma excelsa *crude extract	RA = 0.380		
	*Cecropia peltata *crude extract	RA = 0.371		
	*Rivina humilis *crude extract	RA = 0.371		
	*Mimosa pudica *crude extract	RA = 0.366		
	*Ambrosia hispida *crude extract	RA = 0.346		
	*Rhytidophyllum tomentosum *crude extract	RA = 0.242		
	*Stachytarpheta jamaicensis *crude extract	RA = 0.235		
	*Cucurbita pepo *crude extract	RA = 0.234		
	*Ricinus communis *crude extract	RA = 0.225		
	*Azadirachta indica *crude extract	RA = 0.213		
	*Chenopodium ambrosioides *crude extract	RA = 0.206		
	*Allium sativum *crude extract	RA = 0.205		
	*Centrostachys indica *crude extract	RA = 0.205		
	*Annona squamosa *crude extract	RA = 0.203		
	*Aloe vulgaris *crude extract	RA = 0.196		
	*Andrographis paniculata *crude extract	RA = 0.191		
	Eryngial (*trans*-2-dodecenal)	LD50 at 24 h = 0.461 mM LD50 at 48 h = 0,411		

^*∗*^LD50  : median lethal dose; IC50: half-maximal inhibitory concentration; RA: relative activity (RA) LT50 of the most effective extract (shortest time to kill 50%) divided by the LT50 of each extract.

**Table 4:** Drugs/compounds used in in vivo assays against *Strongyloides* spp.

Specie	Animal model	Compound	Anti-*Strongyloides *activity	Parasite load reduction	Dosage	Reference
*S. venezuelensis*	CD1 mice	2-(Butylamino) hexadecan-1-ol	Yes	56%	20 mg/kg/day for 5 days, 1 day after infection	[16]
		2-(Ethylamino) hexadecan-1-ol	No	45%		
		*tert-*Butyl *N*-(1-aminododecan-2-yl) carbamate	No	21%		
		*tert*-Butyl *N*-(1-aminohexadecan-2-yl) carbamate	No	50%		

*S. ratti*	Wistar rats	Monepantel (AAD 1566)	No	0%	32 mg/kg	[27]

*S. venezuelensis*	Wistar rats	*Piper tuberculatum *extract	No	Not shown	150 mg/kg	[28]
	Wistar rats	*Lippia sidoides *essential oil	Yes	74.4%	150 mg/kg	

*S ratti*	Wistar rats	Tribendimidine	Yes	98.9%	50 mg/kg 72 h after infection	[17]
